# Relationship Between GLP-1-Based Therapies and Periodontal Health: A Systematic Review of Current Evidence and Future Perspectives

**DOI:** 10.3390/ijms27146447

**Published:** 2026-07-20

**Authors:** Kacper Nijakowski, Dawid Gruszczyński, Szymon Łacinik, Jakub Zdrojewski, Livia Ottolenghi, Marta Mazur

**Affiliations:** 1 Department of Conservative Dentistry, Poznan University of Medical Sciences, 60-812 Poznań, Polandjzdrojewski@ump.edu.pl (J.Z.); 2 Department of Oral and Maxillofacial Sciences, Sapienza University of Rome, 00161 Rome, Italy; livia.ottolenghi@uniroma1.it; 3 Department of Endocrinology, Metabolic Disorders and Internal Medicine, Poznan University of Medical Sciences, 60-355 Poznań, Poland; dawid.j.gruszczynski@gmail.com; 4 Interdisciplinary Department of Wellbeing, Health and Environmental Sustainability (BeSSA), Sapienza University of Rome, 02100 Rome, Italy; marta.mazur@uniroma1.it

**Keywords:** glucagon-like peptide-1 receptor agonists, GLP-1, periodontal disease, periodontitis, obesity, diabetes mellitus, periodontium, periodontal therapy

## Abstract

Glucagon-like peptide-1 receptor agonists (GLP-1RAs), widely used in the management of type 2 diabetes mellitus and obesity, have recently attracted attention for their potential effects on periodontal tissues. This systematic review aimed to evaluate the current evidence regarding the relationship between GLP-1-based therapies and periodontal health, with particular emphasis on anti-inflammatory, osteogenic, and regenerative mechanisms. A comprehensive literature search of PubMed, Web of Science, and Embase databases identified 22 eligible studies, including in vitro, animal, and human investigations. The available evidence suggests that GLP-1RAs such as liraglutide and exendin-4 may attenuate periodontal inflammation, reduce alveolar bone loss, and enhance osteogenic differentiation of periodontal ligament and dental pulp stem cells through modulation of pathways including MAPK/ERK, Wnt/β-catenin, NF-κB, and PKCβ2. Clinical observations additionally indicate a bidirectional relationship between periodontitis and incretin signalling, with periodontal therapy associated with increased systemic GLP-1 levels. However, the current evidence remains heterogeneous and is largely limited to preclinical and observational studies. Randomised clinical trials are required to determine the clinical efficacy and therapeutic relevance of GLP-1-based therapies in periodontitis management.

## 1. Introduction

Periodontitis is one of the most prevalent chronic inflammatory diseases worldwide and is increasingly recognised as a systemic condition associated with multiple metabolic disorders, including obesity and type 2 diabetes mellitus (T2DM). Growing evidence indicates that the relationship between periodontal inflammation and metabolic homeostasis is bidirectional, prompting increasing interest in therapeutic strategies capable of simultaneously targeting both conditions [1,2]. In response to these metabolic disorders, glucagon-like peptide-1 receptor agonists (GLP-1RAs) have been introduced as a modern pharmacological intervention representing a pinnacle of rational drug design [3].

These agents are engineered to resist enzymatic degradation by dipeptidyl peptidase-4 (DPP-4), providing sustained therapeutic action through various formulations [4,5]. The primary documented effects of GLP-1RAs include significant improvements in glycaemic control, substantial reduction in body weight via appetite suppression, and robust cardioprotective benefits in high-risk populations [4,6,7]. Current clinical guidelines firmly position GLP-1RAs as a first-line consideration for adults with T2DM and established cardiovascular disease [8]. Beyond these established metabolic roles, an increasing body of research suggests that GLP-1RAs exert pleiotropic anti-inflammatory and regenerative actions across various organ systems [9,10].

A critical area where these pleiotropic effects may be relevant is periodontitis, a prevalent chronic inflammatory disease that is now recognised as a systemic condition [5,11]. Recent systematic evaluations of epidemiological data from the last decade estimate that approximately 62% of dentate adults are affected by some form of periodontitis [12]. The modern classification framework for periodontitis incorporates biological staging and grading to better address the complexity of patients where oral inflammation is a primary modifiable contributor to the overall systemic inflammatory burden [13]. There is a well-established bidirectional relationship between periodontal health and T2DM [5]. Chronic hyperglycaemia exacerbates periodontal tissue destruction through mechanisms such as lipotoxicity and increased oxidative stress [14,15]. Conversely, untreated periodontitis can further impair metabolic control by promoting systemic low-grade inflammation and increasing insulin resistance [5,16]. Recent clinical data even suggest that periodontal inflammation may directly impair the incretin axis, as periodontopathic bacteria like Porphyromonas gingivalis produce enzymes that mimic human DPP-4 and degrade endogenous GLP-1 [11,17]. By increasing GLP-1 levels, GLP-1 RAs may counteract these bacterial enzymes while enhancing the host’s capacity to sustain a balanced oral microbiome [18].

**Figure 1:**
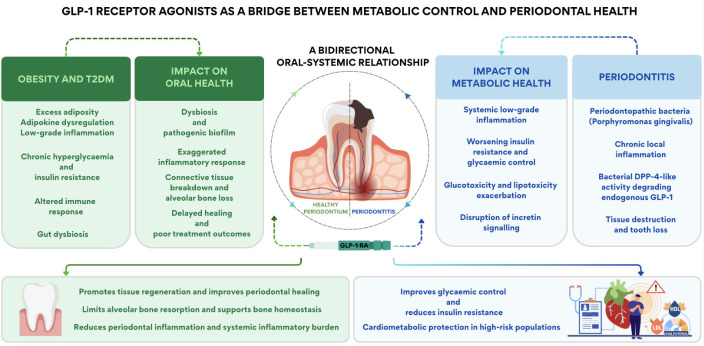
Summary of GLP-1RA impact on the bidirectional oral-systemic relationship between metabolic control and periodontal health.

Current evidence suggests that GLP-1RAs may effectively bridge this oral–systemic gap by reducing periodontal inflammation and limiting alveolar bone resorption [18]. Preclinical findings demonstrate that these agents can enhance the osteogenic differentiation of stem cells and support tissue regeneration, even under high-glucose or inflammatory conditions [11]. These effects are mediated through several potential molecular mechanisms, including the activation of the MAPK/ERK and Wnt/β-catenin pathways, alongside the inhibition of the NF-κB and PKCβ2 signalling cascades [5,18]. Despite these promising insights, it is important to emphasise that current data are predominantly derived from preclinical animal models and observational studies. Consequently, clinical findings remain heterogeneous, and large-scale randomised controlled trials are required to definitively establish the therapeutic efficacy of GLP-1RAs in human periodontal management [16,19]. GLP-1RA’s impact on the bidirectional oral-systemic relationship between metabolic control and periodontal health is summarised in Figure 1.

Several narrative and scoping reviews have recently summarised the potential links between GLP-1 receptor agonists and periodontal health [5,11,18]. However, to the best of our knowledge, no previous systematic review has comprehensively synthesised evidence from in vitro, animal, and human studies. This systematic review aimed to critically evaluate the available evidence regarding the effects of GLP-1 receptor agonists and related incretin-based therapies on periodontal health, including their anti-inflammatory, osteogenic, and regenerative potential, as well as the influence of periodontal disease and periodontal therapy on incretin signalling.

## 2. Methods

### 2.1. Search Strategy and Data Extraction

This systematic review was conducted in line with the Preferred Reporting Items for Systematic Reviews and Meta-Analyses (PRISMA) guidelines [20]—Table S1—and included studies published to 30 April 2026. Literature searches were performed in the PubMed, Web of Science, and Embase databases using predefined search strategies tailored to each database.

The following search terms were applied:

▪ PubMed: (“GLP-1 receptor agonist” OR GLP-1 OR GLP-1RA OR “glucagon-like peptide-1 receptor agonist” OR “GIP receptor agonist” OR “glucose-dependent insulinotropic polypeptide” OR liraglutide OR semaglutide OR dulaglutide OR tirzepatide) AND (“periodontal disease” OR periodontitis OR periodontium)
▪ Web of Science: TS = ((“GLP-1 receptor agonist” OR GLP-1 OR GLP-1RA OR “glucagon-like peptide-1 receptor agonist” OR “GIP receptor agonist” OR “glucose-dependent insulinotropic polypeptide” OR liraglutide OR semaglutide OR dulaglutide OR tirzepatide) AND (“periodontal disease” OR periodontitis OR periodontium))
▪ Embase: (‘GLP-1 receptor agonist’ OR GLP-1 OR GLP-1RA OR ‘glucagon-like peptide-1 receptor agonist’ OR ‘GIP receptor agonist’ OR ‘glucose-dependent insulinotropic polypeptide’ OR liraglutide OR semaglutide OR dulaglutide OR tirzepatide) AND (‘periodontal disease’ OR periodontitis OR periodontium).

Two reviewers independently screened titles, abstracts, and full texts, with disagreements resolved through discussion. Studies were selected according to predefined inclusion criteria based on the PI(E)COS framework (“Population”, “Intervention”, “Comparison”, “Outcomes” and “Study design”), presented in Table 1. The review protocol was prospectively registered in PROSPERO under registration number CRD420261397013. No artificial intelligence tools were used for literature screening, study selection, data extraction, scientific content generation, evidence synthesis, interpretation, or any methodological or scientific decision-making. Artificial intelligence tools were used exclusively for post-writing linguistic refinement (grammar and stylistic editing), as disclosed in the Acknowledgements [21].

**Figure 2:**
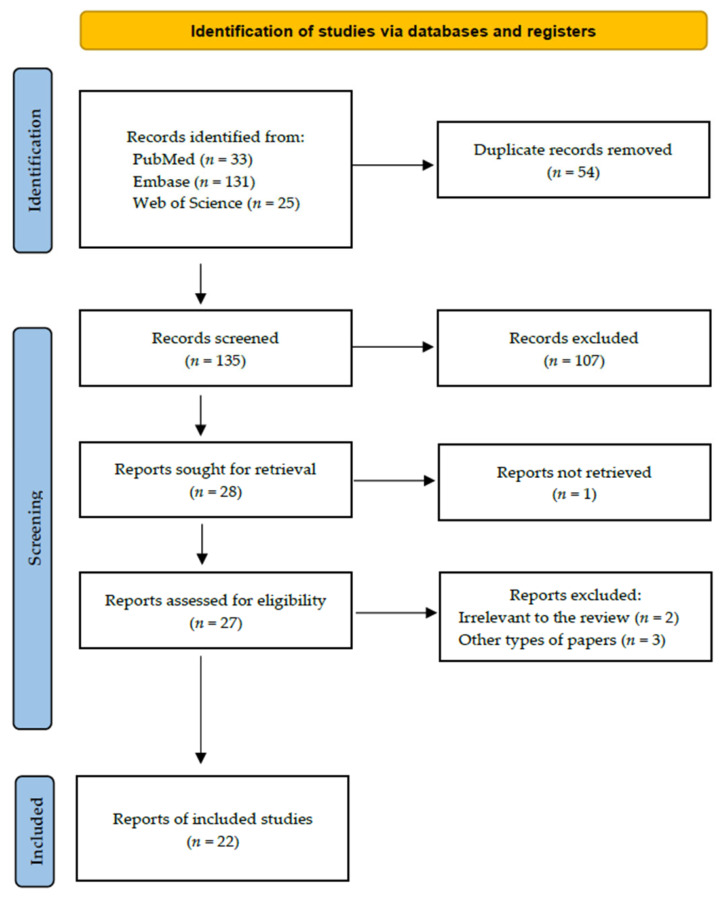
PRISMA flow diagram presenting search strategy.

A total of 22 studies met the eligibility criteria and were included in the review. The complete study selection procedure is presented in Figure 2. The available evidence was highly heterogeneous with respect to study design, investigated GLP-1RAs, experimental models, treatment duration, outcome measures, and study populations. Consequently, quantitative meta-analysis was considered inappropriate. A qualitative narrative synthesis was performed for all included studies, with particular emphasis on the limitations of the available evidence.

### 2.2. Quality Assessment of Included Studies

Risk of bias for each included study was evaluated using the Study Quality Assessment Tool developed by the National Heart, Lung, and Blood Institute of the National Institutes of Health [22] that applies to the observational studies and was adapted for the experimental studies. Assessments were carried out independently by two reviewers, and discrepancies were resolved through discussion. No attempts were made to contact study authors when information was unclear or missing from the published reports.

The results of the quality assessment are summarised in Figure S1. The most common sources of bias included lack of blinding and sample size justification. For critical appraisal, each risk criterion was scored as follows: 1 point for low risk, 0.5 points for unclear risk, and 0 points for high risk. Based on the overall scores, seven studies (31.8%) were rated as having “good” methodological quality (≥85% of the maximum score), whereas fifteen studies (68.2%) were categorised as having “intermediate” quality (≥65% of the maximum score).

According to the Oxford Centre for Evidence-Based Medicine five-level hierarchy [23], all included studies were classified as level III evidence.

## 3. Results and Discussion

### 3.1. Key Findings

Table 2 summarises the main findings from the included studies. Preclinical and early clinical evidence indicates that GLP-1 receptor agonists (GLP-1RAs) may positively modulate periodontal health by reducing inflammation and enhancing bone regeneration. In vitro studies consistently show that GLP-1RAs (exendin-4, liraglutide, etc.) counteract the inhibitory effects of high glucose and inflammation on periodontal ligament and pulp stem cells, promoting proliferation and osteogenic differentiation [24]. Animal models often demonstrate reduced inflammatory markers and attenuated alveolar bone loss with GLP-1RA treatment [25,26,27], though some studies found no bone-sparing effect despite reduced cytokines [19]. Cross-sectional and interventional human data reveal that periodontitis is associated with a dysregulated incretin profile (lower GLP-1, higher glucagon) [17], whereas effective periodontal therapy elevates systemic GLP-1/GIP levels [16]. Mechanistically, GLP-1RAs activate the MAPK/ERK and Wnt/β-catenin pathways and inhibit NF-κB and PKCβ2 signalling, with consistent findings across models [28]. Future research should include randomised clinical trials in diabetic and non-diabetic patients (endpoints: pocket depth, bone density, HbA1c, GLP-1 levels), advanced mechanistic studies (single-cell RNA-seq, proteomics), and improved animal models with glycaemic control.

### 3.2. Effect of GLP-1RAs in Periodontitis

Multiple in vitro studies on human periodontal cells report that GLP-1R agonists enhance regenerative functions. Guo et al. [29] showed in human periodontal ligament stem cells (PDLSCs) cultured under high-glucose conditions that Exendin-4 reversed glucose-induced inhibition of proliferation and osteoblastic differentiation. Mechanistically, Exendin-4 upregulated osteogenic markers (ALP, Runx2, Osx) and activated MAPK signalling (p38, ERK, JNK) and Wnt/β-catenin (β-catenin, p-GSK3β, LEF) [24]. Xu et al. [30] demonstrated that Exendin-4 mitigates LPS-induced premature senescence in PDLSCs via the SIRT1/Notch1 pathway. Liu et al. [31] observed that Exendin-4 suppresses LPS-driven NF-κB activation and boosts Wnt signalling in PDLSCs, favouring osteogenesis. GLP-1 reversed the inhibitory effects of advanced glycation end products (AGEs) on the osteogenic differentiation of human periodontal ligament stem cells by increasing the expression of osteogenic markers, including ALP, BSP, OPN, and Runx2 [32]. Mechanistically, GLP-1 attenuated AGE-induced upregulation of RAGE and PKCβ2 phosphorylation, suggesting that it restores osteogenic potential through inhibition of the PKCβ2 signalling pathway.

Similarly, Pang et al. [33] and Zhang et al. [34] found that liraglutide promoted proliferation, migration, and osteogenic differentiation of human PDL cells while inhibiting inflammatory cytokines, suggesting that it can be used as a potential drug for the treatment of periodontitis. Li et al. [35] reported that liraglutide enhances osteogenesis of dental pulp stem cells (DPSCs) through an epigenetic mechanism: increased histone H3K18 lactylation leading to activation of the DSPP gene. Liraglutide also promoted osteogenic differentiation of human alveolar bone marrow mesenchymal stem cells through activation of the BMP2/Smad/Runx2 signalling pathway [36].

In summary, in vitro evidence consistently shows that GLP-1RAs stimulate anabolic signalling (MAPK/ERK, Wnt/β-catenin), suppress catabolic/inflammatory pathways (NF-κB, PKCβ2), and enhance stem cell functions critical for periodontal regeneration. Unfortunately, the in vitro studies often use supraphysiological agonist doses and do not address chronic infection context. These studies inform mechanisms but not clinical outcomes.

In animal models of periodontitis, GLP-1RAs generally reduced inflammation and supported bone preservation. Sawada et al. [25] showed that systemic liraglutide treatment in ligature-induced periodontitis rats decreased M1 macrophage infiltration and pro-inflammatory cytokines, resulting in significantly less alveolar bone loss. Liraglutide therapy also decreased the number of osteoclasts on the alveolar bone surface. In a study by Yang et al. [37], liraglutide treatment in diabetic rats with periodontitis reduced periodontal inflammation, lowered pro-inflammatory cytokine levels (IL-6, TNF-α, and IL-1β), and decreased alveolar bone resorption while improving alveolar bone microstructure. Additionally, liraglutide enhanced osteogenic activity by decreasing the RANKL/OPG ratio and increasing ALP and Runx2 expression, suggesting its potential as an adjunctive therapy for diabetes-associated periodontitis. Wang et al. [36] observed that, in diabetic and normoglycemic rat models, liraglutide enhanced new bone formation and implant–bone integration, indicating its potential to improve osseointegration under diabetic conditions.

Liang et al. [26] combined SDF-1 and exendin-4 in a rat periodontitis model, observing, in micro-CT, enhanced periodontal bone regeneration compared to controls, consistent with their in vitro PDLSC findings. Combination therapy exerted additive effects on PDLSC proliferation, migration, alkaline phosphatase (ALP) activity, mineral deposition, and the expression of osteogenesis-related genes. Transcriptomic analysis showed that the synergistic effects were associated with regulation of metabolism-, biosynthesis-, immune-, and autophagy-related signalling pathways, as well as amplification of T2DM and insulin-related signalling mechanisms [38]. Pang et al. [27] delivered liraglutide via a PLGA/HA hydrogel in diabetic periodontitis rats; this markedly reduced gingival TNF-α and IL-1β, and suppressed osteoclast-related necroptosis markers (RIPK1/3, MLKL). Therefore, this topical application for four weeks decreased inflammatory cell accumulation in periodontal tissues, mitigated alveolar bone resorption and improved alveolar bone microarchitecture.

In contrast, Moraes et al. [19] reported that exenatide (GLP-1RA) and sitagliptin (DPP-4 inhibitor) lowered periodontal IL-1β, NOS2 and MMP-9 gene expression but did not prevent alveolar bone loss, suggesting some models may show inflammation reduction without bone gain (possibly due to short treatment or strong local ligature insult). The potential role of the DPP-4-like enzyme is consistent with previous reviews, which identify this bacterial enzyme as a biologically plausible mechanism linking periodontitis with altered incretin signalling [11,18]. Although the DPP-4-mimicking activity of *P. gingivalis* may represent an important link between periodontal inflammation and GLP-1 metabolism, its clinical significance remains to be confirmed in human studies.

Additional studies in mice and zebrafish scale models also suggest that incretins may protect periodontal tissues [39,40]. In mice, GIP reduced lipopolysaccharide-induced TNF-α and nitric oxide synthase gene expression in a dose-dependent manner. In a zebrafish regeneration model, treatment with liraglutide enhanced osteoblast differentiation in hDPSCs by increasing calcium deposition, ALP activity, and the expression of osteoblast marker genes, including Runx2, type I collagen, osteonectin, and osteocalcin. Jung et al. [41] noted that exendin-4 ameliorated D-galactose-induced hyposalivation in aging rats, preserving the typical morphological characteristics of acinar cells, reducing both acidic and neutral mucin accumulation, and decreasing apoptotic cells in the salivary gland. It restores oral homeostasis, thereby reducing the risk of dental caries, periodontal diseases, and oral infections.

Rodent models may not fully mimic human periodontitis. Also, dosing routes (systemic vs. local) vary and some studies lack sham controls. Overall, animal evidence supports anti-inflammatory and bone-preserving effects of GLP-1RAs, but translational gaps remain, and consistent bone protection is not universally observed.

### 3.3. Effect of Periodontitis on GLP-1 Levels

Cross-sectional clinical data suggest periodontitis is associated with dysregulated incretin hormones. As noted, Mohamed et al. [42] found significantly higher GCF levels of GLP-1 and GIP in diabetic patients with chronic periodontitis compared to subjects with T2DM without periodontitis. In contrast, in non-diabetic patients with chronic periodontitis, GLP-1 levels were lower than those without periodontal disease. Moreover, Solini et al. [17] reported significant systemic GLP-1 suppression in severely obese, non-diabetic patients with periodontitis. In these patients, after adjustments, probing pocket depth was inversely correlated with GLP-1 levels. Combined with Shen et al.’s findings of downregulated GLP1R/GIPR in PDLSCs from periodontitis sites, the evidence supports that periodontitis is linked to lower GLP-1 signalling [28]. A retrospective survey of 150 periodontitis patients revealed that those on long-term GLP-1R (or dual GLP1R/GIPR) agonist therapy had a significantly better periodontal status (lower clinical staging) than untreated patients. The bidirectional nature of periodontal disease worsening metabolic hormones is biologically plausible via systemic inflammation.

Limitations include the cross-sectional design (causality unclear), confounding metabolic factors, and relatively small sample sizes. Nevertheless, these data imply that periodontitis may impair incretin signalling, and conversely that incretin therapy associates with improved clinical periodontitis outcomes in humans.

### 3.4. Effect of Periodontal Therapy on GLP-1 Levels

Clinical intervention studies show that periodontal treatment can improve incretin levels. Suvan et al. [16] conducted a cohort study in both obese and non-obese periodontitis patients undergoing intensive non-surgical periodontal therapy. They found that serum GLP-1 and GIP levels significantly increased 6 months after therapy in both groups. Notably, subjects with obesity showed a faster rise in GLP-1. This suggests that reducing periodontal inflammation restores a healthier incretin response. Although periodontal therapy has been associated with increased circulating GLP-1 concentrations, these observational findings do not establish causality and may be influenced by residual confounding, including metabolic status, lifestyle modifications, and concomitant pharmacological treatment. However, the effect on GLP-1 in animal models of periodontal treatment has not been studied.

Our findings are also consistent with the broader evidence demonstrating that periodontal therapy contributes to improved glycaemic control in patients with diabetes. Improvements in incretin signalling observed after periodontal treatment may represent one of several biological mechanisms linking periodontal therapy with systemic metabolic benefits [43].

### 3.5. Comparative Synthesis of Included Studies

Nearly all studies report reduced periodontal inflammation with GLP-1RA treatment. For example, liraglutide or exendin-4 uniformly lowered IL-1β, TNF-α, IL-6 and iNOS in gingiva or serum. Inflammatory cell infiltration and M1 macrophage markers were suppressed. These findings align with the broad “anti-inflammatory” profile of GLP-1RAs noted in the literature. Most models showed enhanced osteogenesis and reduced bone loss. Liraglutide reversed LPS/high-glucose-induced suppression of osteogenic genes (Runx2, ALP), and an increased alveolar bone volume in vivo. SDF-1 + exendin-4 co-therapy markedly boosted new bone formation in rat defects. A consistent mechanism is upregulation of osteogenic transcription factors, leading to decreased osteoclastogenesis.

Mechanistic studies frequently implicate NF-κB/MAPK inhibition and Wnt/β-catenin modulation. These pathways intersect with known GLP-1R signalling to suppress cytokine release and osteoclast activity. SDF-1 co-delivery synergised with exendin-4 to enhance cell homing and bone regeneration via the ERK signalling pathway. The discussed pathways should not be regarded as independent mechanisms but rather as interconnected components of inflammatory and regenerative signalling networks. Emerging GLP-1/GIP dual agonists (e.g., tirzepatide analogues) showed even stronger bone-anabolic signals (↑SIRT1, BMP signalling) in preliminary models, though data are scant. This suggests potential for combined incretin strategies.

Most studies align on reduced inflammation and bone loss, but a few nuances exist. Mohamed et al. [42] found that while exendin-4 + sitagliptin lowered IL-1β, MMP-9 and iNOS gene expression, it did not significantly prevent alveolar bone loss in ligatured rats. This suggests inflammation attenuation does not always fully translate into bone preservation. Differences in model (e.g., diabetic vs. non-diabetic rats) or drug dosing might explain such discrepancies. Also, human data are limited—observational studies hint at GLP-1–incretin interplay with PD but cannot prove causality.

The conclusions of the present systematic review are generally consistent with previous narrative and scoping reviews by Jeong et al. [11], Ahmad et al. [18], and Polymeri et al. [5], all of which proposed anti-inflammatory and regenerative effects of GLP-1RAs in periodontal tissues. However, unlike previous reviews, the present study systematically appraised the available evidence and demonstrated that current conclusions are based predominantly on preclinical and observational studies. Consequently, the overall certainty of evidence remains limited despite the biological consistency observed across studies.

The methodological quality assessment should also be considered when interpreting the findings. Most included studies were rated as having intermediate methodological quality, mainly due to the lack of blinding, insufficient sample size justification, and limited reporting of allocation procedures. Consequently, while the available evidence consistently supports the anti-inflammatory and osteogenic potential of GLP-1-based therapies, the magnitude of these effects should be interpreted with caution. The overall consistency of the findings across different experimental models strengthens their biological plausibility; however, the current level of evidence remains insufficient to establish definitive clinical recommendations. Well-designed randomised controlled trials with standardised periodontal and metabolic outcomes are therefore required to validate the promising preclinical findings and determine their translational relevance in routine clinical practice.

### 3.6. Study Limitations and Future Directions

The reviewed evidence is heterogeneous. Due to the heterogeneity of study designs, the NHLBI assessment tool was pragmatically adapted for experimental studies. This approach should be interpreted with caution because several criteria are not directly applicable to laboratory-based investigations. Most in vitro/animal studies use supra-physiologic GLP-1RA doses and short durations. Animal models vary (ligature-induced vs. LPS-induced vs. diabetic), making comparisons hard. Few studies included sham or placebo controls. In humans, data are mostly observational or uncontrolled cohorts. Metabolic confounders (diabetes, obesity, medications) are often present. Publication bias toward positive findings is possible. Mechanistic studies in cell culture may not reflect the complex biofilm–host interactions of human periodontitis.

Future research should focus on large randomised controlled trials of GLP-1RAs as adjuncts in periodontitis, especially in patients with T2DM or obesity. Trial endpoints should include clinical periodontal measures (pocket depth, attachment gain), radiographic bone changes, and systemic markers (HbA1c, GLP-1/GIP levels, inflammatory cytokines). Subgroup analysis by BMI and glycaemic status would be valuable. Mechanistic research should employ omics approaches.

Another important research gap concerns the absence of large real-world evidence studies evaluating periodontal outcomes among users of GLP-1 receptor agonists. Large electronic health record databases (e.g., TriNetX) have already been successfully used to investigate numerous systemic outcomes associated with GLP-1RAs, yet comparable studies focusing on periodontal disease are currently lacking. Such analyses could provide valuable complementary evidence regarding the long-term effectiveness of these therapies in routine clinical practice.

**Table 2:** Characteristics and main findings from included studies.

Author (Year)	Model	Intervention	Main Outcomes (Inflammation, Bone)	Mechanisms
Guo et al. (2018) [29]	In vitro hPDLSCs (high glucose exposure)	Exendin-4	Reversed hyperglycaemia-induced inhibition of proliferation and osteogenesis	↑ ALP, Runx2, and Osx
Liu et al. (2019) [31]	In vitro hPDLSCs (LPS-induced PD)	Exendin-4	↑ proliferation and osteogenic differentiation	↑ ALP, Runx2, and Osx, ↓ TNF-α and IL-6, ↓ IκBα and p-IκBα, ↓ β-catenin (↓ NF-κB pathway, ↑ Wnt pathway)
Wang et al. (2023) [24]	In vitro hPDLSCs (hyperglycaemia exposure)	Exendin-4	Restored osteogenesis inhibited in high-glucose environment	↑ MAPK (p38/JNK/ERK) and Wnt, ↑ ALP, Runx2, and Osx
Xu et al. (2025) [30]	In vitro hPDLSCs (LPS-induced PD)	Exendin-4	Reversed premature senescence of PDLSCs	↑ SIRT1, ↓ Notch1
Kang et al. (2021) [38]	In vitro hPDLSCs (transcriptomic)	SDF-1 + Exendin-4	Synergistic ↑ proliferation, migration and osteogenic differentiation	RNA polymerase II transcription, cell differentiation, chromatin organisation, and protein phosphorylation pathways; NF-κB and TNF signalling pathway; oxidation reduction process, metabolic process, regulation of transcription, DNA-templated, protein phosphorylation, and lipid metabolic process pathways
Liang et al. (2018) [26]	In vitro hPDLSCs + in vivo rats (ligature-induced PD)	SDF-1 + Exendin-4	In vitro: ↑ proliferation, migration and osteogenic differentiation; in vivo: ↑ new bone formation, ↑ early-stage osteoclastogenesis and osteogenesis in regenerated bone, ↑ migration of CXCR4+ and CD90+/CD34− cells in defect areas	↑ ERK signalling pathway
Li et al. (2026) [35]	In vitro hDPSCs	Liraglutide	↑ proliferation and osteogenic differentiation	↑ H3K18 lactylation; DMP1, DSPP, Runx2, OCN and OPN
Pang et al. (2019) [33]	In vitro hPDLCs (LPS-induced)	Liraglutide	↑ proliferation, migration and formation of mineralisation nodes	↑ ALP and Runx2, ↓ TNF-α and IL-6 (↓ NF-κB)
Wang et al. (2024) [36]	In vitro hBMSCs (diabetic) + in vivo (rats—implantation)	Liraglutide	In vitro: ↑ osteogenesis and migration; in vivo: ↑ new bone formation and osseointegration	↑ BMP2/Smad/Runx2 signalling
Zhai et al. (2023) [40]	In vitro hDPSCs + zebrafish scale regeneration model	Liraglutide	In vitro: ↑ osteoblast differentiation of hDPSCs; in vivo: ↑ bone formation; ↑ calcium deposition, ALP activity, osteoblastic cell count, collagen 1α, osteonectin, osteocalcin	↑ Runx2/LncRNA-LINC00968/miR-3658 signalling
Zhang et al. (2020) [34]	In vitro hPDLCs (LPS-induced PD) + in vivo (rat ligature-induced PD)	Liraglutide	In vitro: ↑ osteogenesis; in vivo: ↓ inflammatory cell infiltration in periodontal tissues, ↓ serum TNF-α, IL-1β, and IL-6, ↓ alveolar bone resorption	↑ ALP and Runx2, ↓ Wnt/β-catenin pathway
Wang et al. (2020) [32]	In vitro hPDLSCs (AGEs exposure)	GLP-1	↑ osteogenic differentiation, reversed AGE-induced inhibition of proliferation	↓ RAGE and pPKCβ, ↑ ALP, BSP, OPN, and Runx2
Suzuki et al. (2016) [39]	Mouse GIPRKO (ligature-induced PD) + in vitro THP-1 cells (LPS-induced PD)	GIP	GIPRKO: ↑ inflammatory cell infiltration, ↑ macrophage accumulation	↓ TNF-α and iNOS, partially cAMP/PKA pathway
Jung et al. (2023) [41]	Rat (D-galactose model of aging)	Exendin-4	↑ salivary secretion, ↓ acidic and neutral mucin accumulation, ↓ apoptotic cells in the salivary gland	-
Moraes et al. (2015) [19]	Rat (ligature-induced PD)	Exenatide (GLP-1 agonist), Sitagliptin (DPP-4 agonist)	No change in alveolar bone loss	↓ IL-1β, NOS2, MMP-9 gene expression
Pang et al. (2025) [27]	Rat (diabetic + ligature-induced PD)	Liraglutide (topical PLGA/HA)	↓ TNF-α and IL-1β in GCF and serum, ↓ inflammatory cell infiltration in periodontal tissues, ↓ alveolar bone resorption, ↑ alveolar bone microstructure	↓ RIPK1, RIPK3 and MLKL (necroptosis-related factors)
Sawada et al. (2020) [25]	Rat (ligature-induced PD)	Liraglutide	↓ macrophage M1; ↓ alveolar bone resorption and osteoclasts on the alveolar bone surface	↓ TNF-α and iNOS (↓ NF-κB pathway)
Yang et al. (2022) [37]	Rat (diabetic PD)	Liraglutide	↓ glycemia, serum IL-6, TNF-α, and IL-1β; ↓ alveolar bone resorption, ↑ microstructure of alveolar bone, and ↓ periodontal inflammation	↓ NF-κB pathway, ↑ ALP and Runx2
Solini et al. (2019) [17]	Human (cross-sectional; obesity, PD+/−)	–	PD: ↓ GLP-1, ↑ glucagon and GIP; PPD positively correlated to glucagon and inversely to GLP-1	–
Suvan et al. (2021) [16]	Human (cohort; PD, obesity+/−)	Periodontal treatment	After 6 months, ↑ serum GLP-1 and GIP in obese and non-obese groups, ↑ GLP-1 more rapidly in obese individuals	–
Mohamed et al. (2015) [42]	Human (cross-sectional; T2DM+/−, PD+/−)	–	In GCF of T2DM with PD: ↑ GLP-1 and GIP vs. T2DM without PD	–
Shen et al. (2026) [28]	In vitro hPDLSCs + in vivo (mouse + human survey)	GLP1R/GIPR agonists	In vitro: GLP1R agonist: ↑ differentiation, GIPR agonist: ↑ proliferation; in mice, ↑ PDLSC-mediated periodontal regeneration; patients on GLP1R agonists have better periodontal staging and grading (longer agonist exposure, greater improvement)	↑ MAPK/ERK; ↓ IFIT1–3 by GLP1R signalling

Legend: hPDLSCs, human periodontal ligament stem cells; hDPSCs, human dental pulp stem cells; hBMSCs, human alveolar bone marrow mesenchymal stem cells; PD, periodontal disease; LPS, lipopolysaccharide; AGEs, advanced glycation end products; GIPRKO, glucose-dependent insulinotropic polypeptide receptor knockout; T2DM, type 2 diabetes mellitus; GCF, gingival crevicular fluid; GLP-1, glucagon-like peptide-1; GIP, glucose-dependent insulinotropic polypeptide; DPP-4, dipeptidyl peptidase-4; ALP, alkaline phosphatase; TNF-α, tumour necrosis factor alpha; IL, interleukin; PPD, probing pocket depth; MMP, matrix metalloproteinase.

## 4. Conclusions

Current evidence suggests that GLP-1-based therapies may exert beneficial effects on periodontal tissues by reducing inflammation, promoting osteogenic differentiation, and limiting alveolar bone loss. Experimental studies consistently demonstrate modulation of key inflammatory and regenerative pathways, while preliminary human data indicate potential clinical benefits and a bidirectional relationship between periodontal status and incretin regulation. Nevertheless, the available evidence is predominantly derived from in vitro, animal, and observational studies, limiting direct clinical translation. Future randomised controlled trials with standardised periodontal and metabolic outcomes are necessary to clarify the therapeutic role of GLP-1RAs as adjunctive agents in periodontal treatment, particularly in patients with diabetes and obesity.

## Figures and Tables

**Table 1:** Inclusion and exclusion criteria according to the PICOS.

Parameter	Inclusion Criteria	Exclusion Criteria
Population	Patients aged 0–99 years, both genders, with periodontitis; animals with induced periodontitis; periodontal cells	Individuals with diabetes and/or obesity, without periodontitis
Intervention	GLP-1 receptor agonists	Other medications
Comparison	Not applicable	-
Outcomes	Changes in periodontal inflammatory status (based on, e.g., periodontal indices, tissue-level outcomes), regenerative properties of periodontal cells	Other oral outcomes based on GLP-1-based therapy
Study design	Case–control, cohort, and cross-sectional human studies; in vitro and animal models	Literature reviews, case reports, expert opinion, letters to the editor, conference reports
	Indexed to 30 April 2026	Not published in English

## Data Availability

No new data were created or analyzed in this study. Data sharing is not applicable to this article.

## Supplementary Materials

The following supporting information can be downloaded at https://www.mdpi.com/article/10.3390/ijms27146447/s1.
