# Supplementary material for: Relationship Between GLP-1-Based Therapies and Periodontal Health: A Systematic Review of Current Evidence and Future Perspectives
Source: Int J Mol Sci. 2026 Jul 20;27(14):6447. doi: 10.3390/ijms27146447 (PMC13411477; doi:10.3390/ijms27146447)
Supplement: Supplementary file 1 [file ijms-27-06447-s001.zip › Figure S1.pdf]

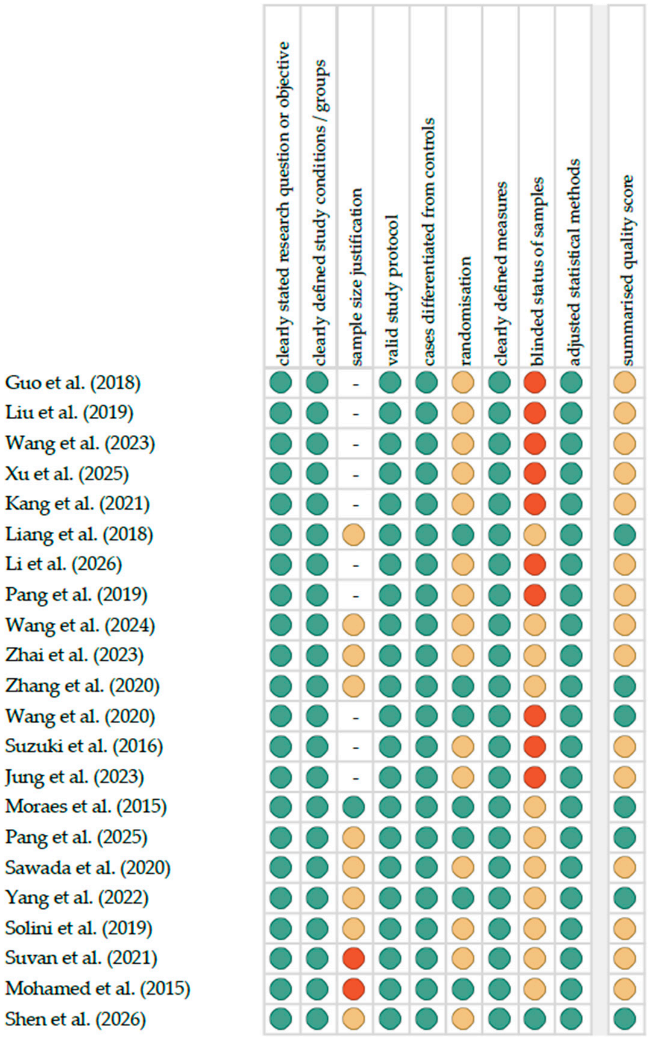

Figure S1. Quality assessment, including the main potential risk of bias (risk level: green—low, yellow—unspecified, red—high; quality score: green—good, yellow—intermediate, red—poor) [16,17,19,24–42].
